# Polydopamine Modified Superparamagnetic Iron Oxide Nanoparticles as Multifunctional Nanocarrier for Targeted Prostate Cancer Treatment

**DOI:** 10.3390/nano9020138

**Published:** 2019-01-22

**Authors:** Nimisha Singh, Fadoua Sallem, Celine Mirjolet, Thomas Nury, Suban Kumar Sahoo, Nadine Millot, Rajender Kumar

**Affiliations:** 1 Department of Applied Chemistry, Sardar Vallabhbhai National Institute of Technology, 395007 Surat, India; nimisha.singh01@gmail.com (N.S.); sks@chem.svnit.ac.in (S.K.S.); 2 Laboratoire Interdisciplinaire Carnot de Bourgogne (ICB), UMR 6303 CNRS/Université Bourgogne Franche-Comté, 21 000 Dijon, France; fadouasallem@gmail.com; 3 Radiotherapy Department, Centre Georges-François Leclerc, 21 000 Dijon, France; CMirjolet@cgfl.fr; 4 Laboratoire Bio-PeroxIL, Université Bourgogne Franche-Comté/Inserm, 21 000 Dijon, France; thomas.nury@u-bourgogne.fr

**Keywords:** core-shell nanoparticles, biocompatible, drug delivery, anticancer

## Abstract

Polydopamine (pDA)-modified iron oxide core-shell nanoparticles (IONPs) are developed and designed as nanovectors of drugs. Reactive quinone of pDA enhances the binding efficiency of various biomolecules for targeted delivery. Glutathione disulfide (GSSG), an abundant thiol species in the cytoplasm, was immobilized on the pDA-IONP surface. It serves as a cellular trigger to release the drug from the nanoparticles providing an efficient platform for the drug delivery system. Additionally, GSSG on the surface was further modified to form S-nitrosoglutathione that can act as nitric oxide (NO) donors. These NPs were fully characterized using a transmission electronic microscopy (TEM), thermogravimetric analysis (TGA), dynamic light scattering (DLS), zeta potential, X-ray photoelectron spectroscopy (XPS), Fourier transform infrared (FTIR) and UV-vis spectroscopies. Doxorubicin (DOX) and docetaxel (DTX) are two anticancer drugs, which were loaded onto nanoparticles with respective loading efficiencies of 243 and 223 µmol/g of IONPs, calculated using TGA measurements. DOX release study, using UV-vis spectroscopy, showed a pH responsive behavior, making the elaborated nanocarrier a potential drug delivery system. (3-(4,5-dimethylthiazol-2-yl)-5-(3-carboxymethoxyphenyl)-2-(4-sulfophenyl) -2H-tetrazolium (MTS) and apoptosis assays were performed on PC3 cell lines to evaluate the efficiency of the developed nanocarriers. These nanoparticles thus can prove their worth in cancer treatment on account of their easy access to the site and release of drug in response to changes to internal parameters such as pH, chemicals, etc.

## 1. Introduction

Multifunctional nanohybrid systems offer several advantages for integrating functional nanocomponents into one entity to manifest different roles in cancer therapeutics. They provide an ideal platform to construct the nanostructure material that can be tuned to the required physical and chemical properties. Although new chemotherapeutic molecules have been continuously developed to treat cancer, medical advancement has failed to control the serious side effects caused by such compounds [1]. Thus, to address these issues and have a targeted anticancer treatment, efficient drug delivery carrier is required. Nano drug delivery carrier shows the potential of carrying the anticancer drug to the targeted site and delivering it according to the required pharmacology activity. Consequently, core shell nanoparticles have emerged as a promising nanocarrier for targeting tumor cells [2].

Uniform core shell nanoparticles systems, having a magnetic core with a functional shell, have been widely explored in protein separation, targeted drug delivery, magnetic resonance imaging, etc. [3,4,5]. Polydopamine (pDA) coated iron oxide nanoparticles (IONPs) are such a class of nanohybrids that have been developed with considerable interest as a multifunctional platform, thanks to their unique biocompatibility, enhanced magnetic resonance (MR) contrast and facile surface modification [6]. pDA is the polymerized structure of dopamine (DA) monomer that undergoes self-polymerization at alkaline pH giving both catechol and amine functional groups on the surface [7]. During the polymerization step, it results in the formation of a conformal and continuous coating layer on any substrate material via strong binding affinity of catechol functional groups. Moreover, this shell thickness can be potentially monitored by varying the reaction condition and the amount of DA in the medium [8].

Recent years have witnessed an exponential growth in research concerning advance functionalization and application of pDA-based nanohybrids in medical fields [9]. Thus, we chose pDA-coated IONPs for immobilizing glutathione disulfide (GSSG), which can serve as an efficient nanocarrier to carry doxorubicin (DOX) and docetaxel (DTX) for prostate cancer treatment. pDA is known to carry highly functional groups on the surface that allows the immobilization of biomolecules via Michael addition [9]. Immobilizing biomolecules on the pDA surface can increase the efficacy of drug release in the system [10].

It was reported that nanocarriers bearing reducible disulfide bonds can be easily cleaved with GSSG by thiol/disulfide exchange [11]. Glutathione in living cells exists in a redox equilibrium state between the disulfide (GSSG, oxidized form) and sulfhydryl (GSH, reduced form) where the change in the concentration of glutathione level marks the change in the oxidative stress caused by the tumor cells. Thus, the triggered release largely depends on the redox potential controlled by the oxidation and the reduction of glutathione (GSH/GSSG) [12] and, therefore, can attain the intracellular delivery of loaded drug [13], DNA [14], and siRNA for cancer therapeutics [15]. Thus, biocompatible nanohybrids with disulfide/thiol groups are of great interest in improving the drug delivery system.

Our objective is focused on immobilizing GSSG on pDA-IONPs to serve as a dual purpose towards prostate cancer treatment. Since GSH plays an important role in determining various cellular processes, it has also been reported that it can be a determining factor for the sensitivity of some tumors to several chemotherapeutic agents [16]. It may also turn out to be a successful biomarker for selecting tumors potentially responsive to chemotherapeutic regimens.

**Scheme 1:**
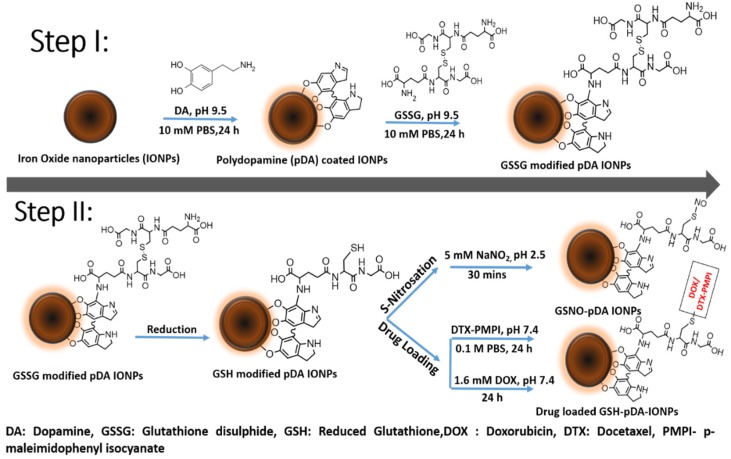
Schematic representation showing the sequential synthesis of Step I: GSSG functionalized pDA-coated IONPs; and Step II: GSSG reduced to GSH-modified pDA-IONPs followed by S-nitrosation and loading of anticancer drugs (DOX and DTX).

Thus, firstly, we chose GSSG to reduce its disulfide bond in order to have thiol groups on IONP’s surface. These free thiol groups can serve as nitric oxide (NO) donors in the system by forming S-nitrosothiols (SNOs). SNOs possess the same physiological functions as NO, which include neurotransmission, hormone secretion and vasodilation in living bodies [17,18]. Additionally, NO has also emerged as antimicrobial agent [19] and has a tumoricidal factor [20] that makes it a promising pharmaceutical agent. Secondly, reduced GSSG (GSH) can carry DOX or DTX drugs for targeted prostate cancer cell treatment. While DOX has been widely used in the treatment of hematological and solid tumor malignancies, DTX is popularly used in the treatment of prostate cancers. Thus, in the present work we explored DOX activity in treating prostate cancer along with DTX. The details of the objective can be better understood with Scheme 1.

## 2. Materials and Methods

Doxorubicin HCl (DOX), dopamine (DA), dithiothreitol (DTT), sodium hydroxide (NaOH, ≥97%), hydrochloric acid (HCl, 37%), nitric acid (HNO_3_, 69%), Elman’s reagent 5-dithiobis[2 nitrobenzoicacid] (DTNB) and ammonium hydroxide (NH_4_OH, 28%) were obtained from Sigma Aldrich (St. Louis, MO, USA). p-maleimidophenyl isocyanate (PMPI) was purchased from Thermo Scientific (Fair Lown, NJ, USA). Iron (II) chloride tetrahydrate (FeCl_2_·4H_2_O, 98%) and iron (III) chloride hexahydrate (FeCl_3_·6H_2_O, 97%) were purchased from Alfa Aesar (Karlsruhe, Germany). Docetaxel (DTX) was purchased from BIOTREND Chemikalien GmbH (Cologne, Germany). PBS 1× solution (Fisher Bioreagents, Fair Lown, NJ, USA), and dimethyl sulfoxide (DMSO, ≥99.7%) (Acroseal) were also purchased from Fisher Chemicals (Leicestershire, UK). Annexin V-FITC (fluorescein isothiocyanate) detection kit with PI (propidium iodide) staining for cell apoptosis was obtained from BD biosciences (Franklin Lakes, NJ, USA). Borate buffered saline was prepared from boric acid (99.8%). Acetate buffer was prepared from sodium acetate and acetic acid. The ultrafiltration stirred cell (Model 8400, 400 mL) and membranes (ref. #PLHK07610, regenerated cellulose 100 kDa) were acquired from Merck Millipore (Darmstadt, Germany). All other chemicals were used of analytical grade and without further purification.

### 2.1. Characterization Techniques

The size and the morphology of the nanoparticles were analyzed using a JEOL JEM-2100 LaB6 transmission electronic microscope (TEM, Tokyo, Japan) with an acceleration voltage of 200 kV and equipped with a high tilt pole-piece achieving a point-to point resolution of 0.25 nm. The samples were prepared by evaporating a diluted suspension of NPs in deionized water on a carbon-coated copper grid. The average size of nanoparticles was determined by counting individual IONPs on each sample (150 nanoparticles).

The chemical composition of the NPs’ surface, involved during the process of nanohybrid synthesis, was investigated with X-ray photoelectron spectroscopy (XPS) using PHI 5000 Versaprobe apparatus (ULVAC-PHI, Osaka, Japan) with a monochromatic Al Kα1 X-ray source (energy of 1486.7 eV with a 200 μm spot size, accelerating voltage of 12 kV, and power of 200 W). The applied pass energy (PE) was 180 eV for the spectra and 50 eV for windows. Powders were pressed on an indium sheet. Data were analyzed using CasaXPS software for processing the peaks. Neutralization was used to minimize charge effects and the carbon C1s peak at 284.5 eV was used as the reference. A Shirley background was subtracted and Gauss (70%)–Lorentz (30%) profiles were used. Full width at half maximum (FWHM) was fixed between 1.5 and 1.9 eV. ULVAC-PHI MultiPak software (ver. 9.0.1, Osaka, Japan) was employed for quantitative analysis.

To confirm the functionalization of pDA and the immobilization of GSSG over the IONPs, a Bruker Vertex 70v (Billerica, MA, USA) using OPUS version 3.1 was used to obtain Fourier transform infrared (FT-IR) spectra using the KBr method with the wavenumber range of 400–4000 cm^−1^, resolution of 4 cm^−1^ and a total of 30 scans per measurement.

UV-visible spectroscopic measurements were carried out to study the absorbance of the as prepared nanoparticles and drug release kinetics using a Shimadzu UV-2550 UV-Vis spectrophotometer (Tokyo, Japan). All the spectra were measured in the range of 220–800 nm and recorded at room temperature using quartz cuvette of 1 cm path length.

Drug kinetics were also analyzed using fluorescence spectroscopy using a Cary Eclipse fluorescent spectrophotometer (Santa Clara, CA, USA) in the emission range of 380–700 nm. The selected excitation and emission slits were 5 and 10 nm, respectively.

Hydrodynamic diameter and zeta potential measurements were carried out using Malvern Nano ZS instrument (Worcestershire, UK) supplied by DTS Nano V7.11 software (Worcestershire, UK). The Smoluchowski equation was used for zetametry measurements. For each measurement, powders were dispersed in 12 mL of NaCl aqueous solution (10^−2^ M). pH titrations were performed using HCl (0.1 M), NaOH (0.1 M) or NaOH (0.01 M) aqueous solutions. Standard deviations were calculated from three measurements performed on the same sample. Samples were analyzed using a backscattering angle (173°). The refractive index of Fe_3_O_4_ NPs is 2.42 and the absorption is equal to 0.029. Hydrodynamic diameters in this paper refer to the Z-average, which is the intensity weighted mean diameter derived from the cumulants analysis. Hydrodynamic diameter was determined by dynamic light scattering (DLS, Malvern, Worcestershire, UK) curves, which were derived from a number distribution calculation process.

The diffraction data were collected at room temperature, using a D8 Advance X-ray diffractometer (Vantack detector) (Billerica, MA, USA), in the 2θ range 10–100°. The Cu K_α1,2_ radiations (λ_α1_ = 1.540598 Å and λ_α2_ = 1.544426 Å) were applied. The data analysis was carried out with Topas^®^ software (Billerica, MA, USA). Le Bail method was used to obtain lattice parameters and mean crystallite size. The phase identification was established by comparison of the diffraction patterns to the ICDD: The International Centre for Diffraction Data Powder Diffraction File Reference.

Powders were analyzed using a Discovery TGA-TA instrument (Newcastle, UK) with a gas (nitrogen:oxygen 80:20) flow rate of 25 mL min^−1^. The applied thermal program was as following: ramp 1 of 20 °C.min^−1^ from 25 to 100 °C; isotherm at 100 °C for 30 min (to remove the remaining moisture) and ramp 2 of 5 °C.min^−1^ from 100 to 800 °C.

Magnetic susceptibility measurements were performed on a Bartington MS3 magneto-susceptometer (Witney, England) at 300 K. A MS2G mono frequency sensor at 1.3 kHz from Bartington (Oxford, England) was used for around 1 mL cells operated at 1.3 kHz.

### 2.2. Preparation of Coated Iron Oxide Nanoparticles

#### 2.2.1. Synthesis of Bare IONPs

Iron oxide nanoparticles (Fe_3_O_4_, IONPs) were prepared by a facile chemical co-precipitation method [21] where FeCl_3_·6H_2_O (34.6 g) and FeCl_2_·4H_2_O (12.7 g), in the molar ratio of 2:1, were dissolved in 1.5 L of water. Then, Fe_3_O_4_ nanoparticles were precipitated quickly by adding ammonia (120 mL, 28%) in the solution at 22 °C. The obtained black suspension was stirred for 2–3 min. The obtained black precipitate was then magnetically decanted and washed thoroughly with deionized (DI) water until the solution reached pH 8. The precipitate was then kept in nitric acid solution (1 mM, pH 3) and dialyzed against nitric acid solution (10 mM) for 48 h. Dialyzed nanoparticles were then centrifuged for 15 min at 20,000 G with deceleration to obtain stable IONPs. The supernatant was then used for further characterizations and surface modification.

#### 2.2.2. Synthesis of Polydopamine-modified IONPs: pDA-IONPs

A quantity of 100 mg of Fe_3_O_4_ (10 mg mL^−1^) was dispersed under continuous stirring in 25 mL of 10 mM DA solution (PBS, 10 mM, pH 9.0) for 24 h at 22 °C. After, polydopamine-modified nanoparticles (pDA-IONPs) were magnetically decanted, washed thoroughly 5 times with ultrapure water using ultrafiltration to remove the non-reacted DA (ensured using UV), and further dispersed in water.

#### 2.2.3. Preparation of GSSG-modified Nanoparticles

GSSG-modified nanoparticles were prepared using 20 mg of pDA-IONPs, which were dispersed in 1 mg mL^−1^ GSSG solution (10 mM PBS, pH 9.4) for 24 h under continuous magnetic agitation (700 rpm) at 22 °C. The GSSG-modified nanoparticles (GSSG-pDA-IONPs) were magnetically separated using magnetic syringe [22], washed with PBS buffer and checked with DTT to ensure the complete removal of unreacted GSSG. DTT reduces the unreacted disulfide, giving a yellow color solution indicating the presence of disulfide group. The resultant nanoparticles were then washed and re-dispersed in PBS.

The above nanoparticles were then reduced to glutathione to get free –SH. Briefly, GSSG modified nanoparticles (800 µL of the suspension) were first mixed with Tris buffer (0.2 M, pH 9), after which, DTT (0.01 M) was added and the reduction of disulfide was allowed to proceed for 1 h at 22 °C. To ensure the reduction, DTNB (0.01 M) in acetate buffer (0.2 M, pH 5) was reacted with the sample and the absorbance was measured at 408 nm.

S-nitrosation was then performed using NaNO_2_ (0.4 mM). The reaction took place when GSH-pDA-IONPs were successively added in NaNO_2_ solution at 37 °C and pH 2.5 for 30 min in the dark. UV-vis spectra were recorded to see the formation of S-nitrosation and the resultant nanoparticles were finally washed with PBS buffer and re-dispersed in PBS.

### 2.3. Drug Loading

For the incorporation of doxorubicin (DOX), the DOX solution (600 µL, 1 mg mL^−1^) was added dropwise with stirring to an aqueous dispersion of GSH-pDA-IONPs (3 mg of particles in 5 mL of water) at 22 °C. Stirring was continued for 24 h to allow the partitioning of the drug into the nanoparticles. The absorbance of the supernatant was measured to determine the loading efficiency of the nanoparticles before and after the reaction. The obtained nanoparticles were then washed with deionized water (DI), magnetically separated using magnetic syringe and resuspended in DI water for further studies. Quantification and release kinetics of loaded drug were performed using UV-vis spectroscopy and TGA.

For the incorporation of docetaxel (DTX), DTX was first activated using PMPI, where prior to loading, DTX was initially dispersed in DMSO and mixed with PMPI (1:4 molar ratio) in borate buffer saline solution (0.1 M, pH 8.5) for 24 h at 22 °C [23]. The resultant solution was then dialyzed against water to eliminate unreacted PMPI. DTX-PMPI was then analyzed using nuclear magnetic resonance NMR and infrared spectroscopy (FTIR), and loaded on GSH-pDA-IONPs in a reaction medium of PBS (0.1 M, pH 7.4) for 24 h at 22 °C. The developed nanoparticles were then magnetically washed, separated and freeze dried for further characterizations.

### 2.4. Release Profile

After purification of DOX-loaded nanoparticles, the dialysis tube filled with DOX-loaded nanoparticles suspension was transferred to a beaker containing 25 mL of phosphate buffer (10 mM) to study the drug release at 37 °C with continuous stirring at 100 rpm. To quantify the drug release, 2 mL of samples were analyzed at different time interval. The amount of released DOX was analyzed with a spectrophotometer at 485 nm. The release was studied in 3 different pH (3.5, 5.4 and 7.4) where it was adjusted with HCl and NaOH. The experiments were performed in triplicate for all samples.

### 2.5. In Vitro Assays

Cytotoxicity was measured on PC-3 cell lines. The cells, at a concentration of 3000 cells/well, were seeded in 96-well plates and incubated at 37 °C in 190 µL of drug-free culture medium (DMEM) with 10% of fetal bovine serum for 24 h before treatment (when cells were at around 20% confluence). The cytotoxicity assays were performed using the following compounds: free drug (DOX or DTX), GSH-pDA-IONPs, and drug loaded GSH-pDA IONPs. Tumor cells were incubated (10 µL of drug on 190 µL of culture medium) with a range of equivalent drug concentrations. After 48 h incubation, cell viability was evaluated using MTS (3-(4,5-dimethylthiazol-2-yl)-5-(3-carboxymethoxyphenyl)-2-(4-sulfophenyl)-2H-tetrazolium) assay (Promega Corporation, Madison, WI, USA) according to Mirjolet et al. [24]. Experiments were performed 6 times and the results were calculated as the mean of the measurements.

### 2.6. Analysis of Cell Proliferation by Flow Cytometry

Flow cytometry was used to analyze cells’ proliferation after their incubation with a DOX-loaded sample, by estimating the proportion of cells in apoptosis. After 24 h of incubation in 6-well plates, PC-3 cells were exposed to GSH-pDA-IONPs and DOX-GSH-pDA-IONPs for 24 h plus a negative control well. After the treatment, a double staining with annexin V-FITC and propidium iodide (PI) was performed with an FITC annexin V apoptosis detection kit obtained from Thermo Scientific (Fair Lown, NJ, USA). The cells were washed twice in PBS without Ca^2+^ and Mg^2+^, trypsinized with 1 mL of trypsine solution 1× and then centrifuged for 5 min at 300 g. After 2 washes with cold PBS, 1 mL of annexin V binding buffer (1×), 10 µL of annexin V-FITC and 10 µL of PI staining solutions were added to each sample. Finally, cells were incubated for 15 min at 37 °C. Cell suspensions were analyzed with a Galaxy flow cytometer (Partec, Görlitz, Germany). Red fluorescence of PI was collected through a 590-nm long-pass filter and green fluorescence was collected through a 520-nm band-pass filter. For each sample, about 10,000 cells were analyzed and the data were treated with FlowJo (Tree Star Inc., Ashland, OR, USA) software.

## 3. Results

**Figure 1:**
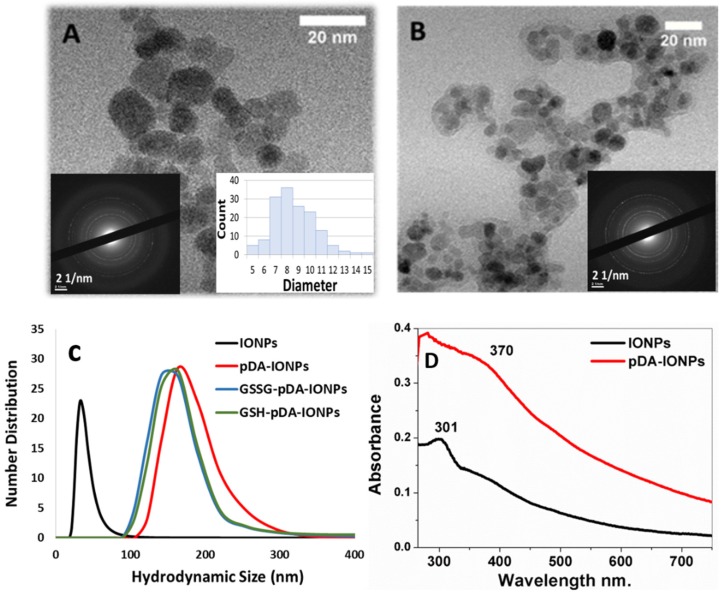
Transmission electronic microscope (TEM) images of (**A**) bare IONPs; and (**B**) pDA-coated IONPs (inset selected area (electron) diffraction (SAED) patterns and size distribution); (**C**) Dynamic light scattering (DLS) measurements showing the hydrodynamic diameter at pH = 5.0–6.0 and in 10^−2^ M NaCl; and (**D**) UV-vis spectra of the synthesized nanoparticles.

The morphology and the size of the synthesized nanoparticles were analyzed using TEM, XRD and DLS measurements as shown in Figure 1, where the size of bare IONPs is calculated to be 9.0 ± 4.0 nm from TEM measurements and 10.1 ± 0.1 nm from XRD (Figure S1). The lattice parameter determined from XRD is 8.368 ± 0.001 Å. The diffracting planes (222), (311), (422) and (511) and the interatomic distances observed on the selected area diffraction pattern of Fe_3_O_4_ (Figures S1 and S2) are in good agreement with the spinel structure (ICDD: 19-0629) [25]. This lattice parameter suggests that the powder is slightly oxidized (Fe_3_O_4_ crystallites and a small presence of γ-Fe_2_O_3_ on the surface) [26,27]. Additionally, with DLS measurements, the hydrodynamic diameter of IONPs at pH 5.0–6.0, is observed to be 33 ± 5 nm (PDI = 0.38 ± 0.07). The TEM image of the polydopamine coated IONPs (Figure 1B) reveals the presence of a homogeneous layer of pDA on the surface with a thickness of about 2 nm, calculated using ImageJ software. It is also observed that even with pDA coating, the crystalline nature of IONPs is retained (inset Figure 1B and Figure S2).

The hydrodynamic diameter, obtained from DLS measurements, is 164 ± 8 nm (PDI = 0.66 ± 0.01) in the case of pDA-IONPs, which is larger than that of bare IONPs. Indeed, pDA form an organic shell with reactive functional groups, which increases the interaction between IONPs and forms small aggregates. This large size can be due to the swelling effect of the polymer when coated over IONPs [28]. During the polymerization step of dopamine, it tends to produce oligomers having from four to eight 5,6-dihydroxyindole units which are assembled in an orderly manner via π stacking to form nanoaggregates [29]. This results in the formation of continuous film on the surface that can be seen in the TEM image (Figure 1B).

UV-vis absorbance spectra are recorded to understand the polymerization step of DA on the surface of IONPs. In Figure 1D, the pDA-IONPs show an absorbance band at 370 nm, which is the characteristic of polymerized layer of quinone, the intermediate molecule of pDA, onto the IONP surface [21]. On the other hand, the characteristic absorbance band of IONPs is observed at 301 nm as shown in Figure 1D.

Since our objective is to have thiol groups on the surface for a wide range of applicability of the developed nanoparticles in the field of medicine, upon binding of GSSG to pDA-IONPs, similar hydrodynamic size (141 ± 2 nm, PDI = 0.68 ± 0.15) is observed. After the reduction of GSSG to GSH, the DLS measurements indicate the same hydrodynamic size as the previous step (140 ± 2 nm, polydispersity index (PDI) = 0.33 ± 0.03). This result shows that reduction step does not affect the hydrodynamic size of coated IONPs.

**Figure 2:**
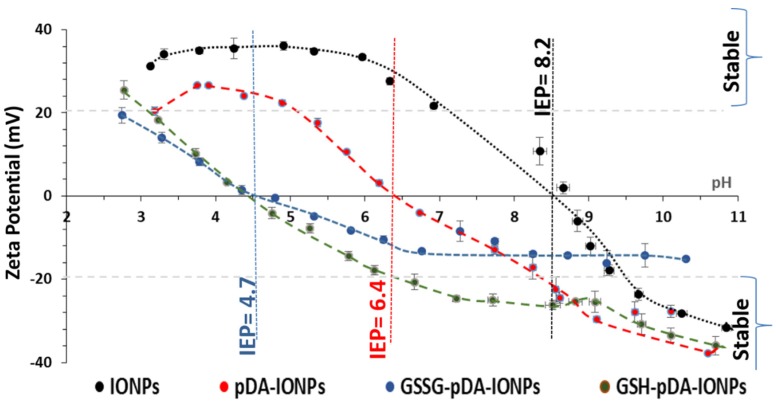
Zeta potential vs pH curves showing the isoelectric point of differently functionalized nanoparticles in 10^−2^ M NaCl.

It has been reported that physicochemical properties such as shape, size and surface charge mark an important role in the cellular uptake of nanoparticles [30]. Indeed, nanoparticles with a negative surface charge show greater cellular uptake due to their high interaction with cells or organs such as the liver [31]. Thus, zeta potential measurements were used to explore the surface charge of coated IONPs. The zeta potential of the synthesized nanoparticles with and without the surface modification with pDA and GSSG as a function of pH is measured as shown in Figure 2. The hydroxyl groups (Fe–OH) on the surface of bare IONPs are responsible for their surface charge. Indeed, in basic condition, it results in negative values of zeta potential due to the formation of Fe–O^−^ and positive values in acidic conditions due to the formation of Fe-OH_2_^+^ [32]. The isoelectric point (IEP) of bare IONPs is measured to be 8.2 which decreases to 6.4, 4.7 and 4.5 upon functionalization with pDA, GSSG and GSH respectively. This shift in the IEP from 8.2 for bare IONPs to 4.5 for GSH-pDA-IONPs suggests the successful immobilization of GSH molecules onto the surface of IONPs [33]. Moreover, the decrease in the zeta potential values, after pDA and GSH grafting, proves the presence of an organic shell which hides a part of the surface charge of IONPs [34]. Also, at physiological pH, the colloidal stability of the synthesized nanoparticles, on the successive molecules’ conjugation, is improved as shown in Figure S3, thanks to the increase in the absolute value of zeta potential and the steric hindrance of the organic molecules, thereby making them suitable nanocarriers for drug loading.

The magnetic nature was also analyzed as a calibration curve shown in Figure S4, supporting information (SI) showing the magnetic susceptibility of the prepared IONPs increases with increase in the concentration of nanoparticles used.

**Figure 3:**
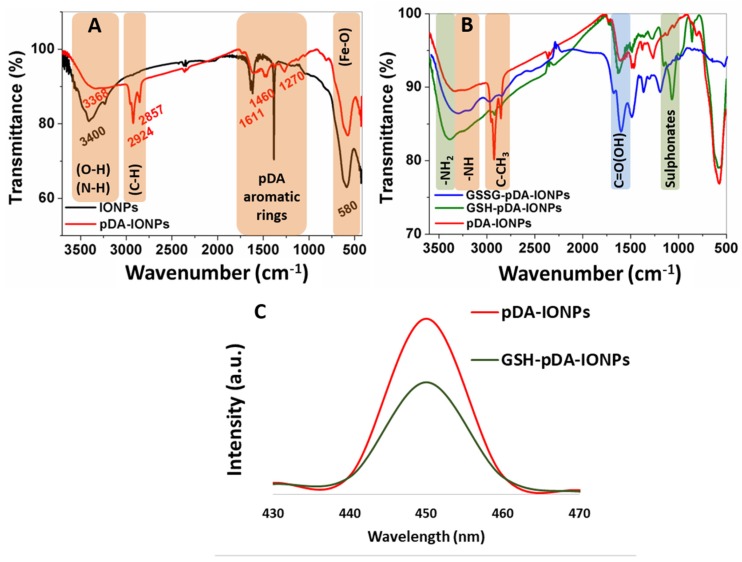
(**A**,**B**) Fourier transform infrared (FTIR) spectra showing the successful conjugation of pDA and GSSG molecules on IONPs; and (**C**) fluorescence spectra of pDA-IONPs and GSH-pDA-IONPs with λ_excitation_ = 400 nm, λ_emission_ = 450 nm.

Chemical and thermal properties of pDA- and GSSG-functionalized IONP_S_ are further characterized using FTIR, XPS and TGA measurements. The presence of pDA layer is confirmed from IR stretching frequencies at 1611, 1460 and 1270 cm^−1^, which correspond to the vibration bands of aromatic rings of pDA (Figure 3A) [21]. Naked IONPs exhibit the magnetite characteristic Fe–O stretching band at 580 cm^−1^, which remains constant even after the formation of a pDA layer on the surface. FTIR spectrum of GSSG-modified pDA-IONPs gives an additional strong stretching band of C=O at 1690 cm^−1^, attributed to the carboxylic group of GSSG, which remains after the reduction of GSSG into GSH (Figure 3B). Furthermore, a broad stretching band of primary amine is observed on GSSG modification at 3300 cm^−1^, which is shifted to 3400 cm^−1^ on the reduction step of GSSG to GSH. This shift may be explained by the intramolecular hydrogen bonding between NH_2_ and carboxylic oxygen. The GSH modified nanoparticles show a slightly quenched fluorescence emission when compared to only pDA-IONPs (Figure 3C), measured at the same concentration, which supports the immobilization step of glutathione onto pDA-IONPs. Indeed, pDA nanoparticles are known to show an inherent fluorescence property [35]. Since pDA possesses reactive quinone and amine groups, it can actively bind to the GSSG molecule and consequently GSH serves as an excellent fluorescent quencher. Thus, GSSG-pDA shows the potential to quench fluorescence via plausible electron-electron transfer energy process [36].

**Figure 4:**
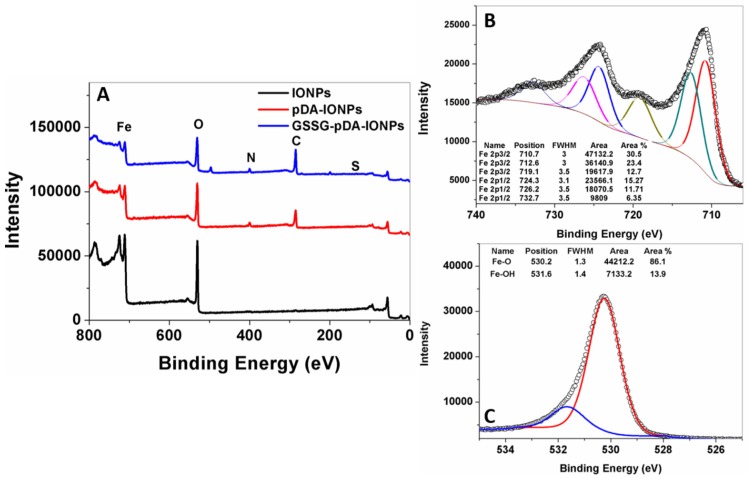
(**A**) X-ray photoelectron spectroscopy (XPS) survey spectra of the prepared nanoparticles with the fitted peaks of (**B**) Fe (2p); and (**C**) oxygen (1s) of bare IONPs.

X-ray photoelectron spectroscopy is a highly surface sensitive technique and provides accurate information about surface properties [37,38]. The XPS survey scans of IONPs, pDA-IONPs and GSSG-pDA-IONPs are shown in Figure 4. The elemental composition, calculated from XPS (Table 1), explains that after pDA coating, the atomic percentages of carbon and nitrogen increase drastically from 3.0% to 41.8% and from 1.1% to 4.5%, respectively, which confirms the presence of a pDA shell, where Fe atomic percentage decreases. Indeed, as the XPS is a surface analysis technique (about 5 nm of depth) and the thickness of organic shell increases progressively after each grafting, the inorganic core becomes more and more covered and not easily accessible. On immobilizing GSSG onto pDA-IONPS, the atomic percentage of carbon (*C*) further increases to 51.1% and a similar trend is observed for nitrogen. This is also observed on analyzing the survey spectra shown in Figure 4A, where the intensity of carbon and nitrogen increase on reacting IONPs with pDA and GSSG.

To understand the nature of the chemical bonds of Fe and O in bare IONPs, decomposition of XPS spectra was realized. On fitting Fe 2p spectrum, it is split into 2p_1/2_ and 2p_3/2_ due to spin orbit j-j coupling as shown in Figure 4B [39]. In addition to the two major peaks (Fe 2p_3/2_ at 710.7 eV and Fe 2p_1/2_ at 724.3 eV), two satellite peaks are also observed, which are Fe(II) 2p_3/2_ satellite peak at 719.1 eV and Fe(III) 2p_1/2_ satellite peak at 732.7 eV (Figure 4B). The oxidation state of iron species present in the sample may be evaluated thanks to these data. Indeed, the binding energy difference between Fe 2p_3/2_ and its satellite peaks is related to the oxidation state of the iron cations; this difference is close to 6 eV for Fe^2+^ and 8 eV for Fe^3+^. In our case, this difference is equal to 8.4 eV for bare IONPs [40]. Thus, XPS shows that the surface of bare IONPs is oxidized with the presence of mainly Fe^3+^. The other contributions, respectively located at 712.6 and 726.2 eV, could not be attributed to Fe^2+^ contribution, because they should be located at lower energies (about 709 eV) [41]. However, they may be assigned to Fe–O–OH [42]. Similarly, the XPS spectrum of oxygen in bare IONPs shows two peaks at 530.2 eV and 531.6 eV, which are attributed to the binding energies of Fe–O–Fe and Fe–OH bonds as shown in Figure 4C [43].

**Figure 5:**
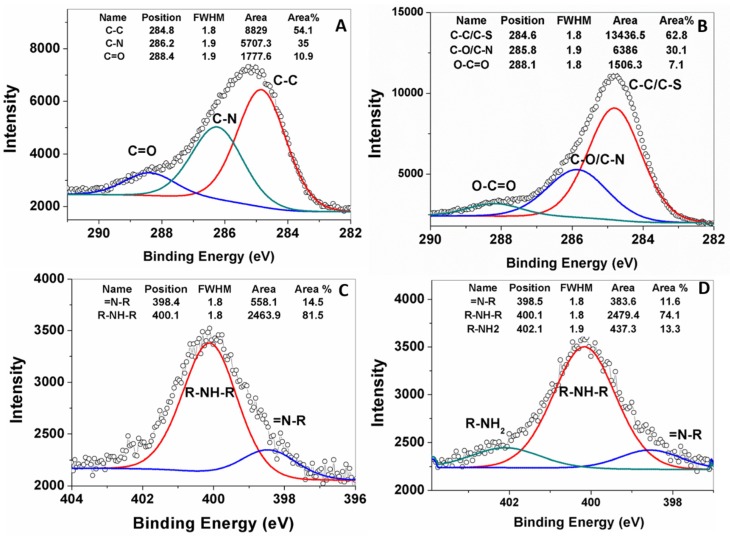
Fitted spectra of C (1s) for (**A**) pDA-IONPs; (**B**) GSSG functionalized pDA-IONPs; and of N (1s) for (**C**) pDA-IONPs; (**D**) GSSG functionalized pDA-IONPs.

XPS peak fittings, performed for pDA-IONPs and GSSG-pDA-IONPs, describe the chemical interaction between the grafted molecules as shown in Figure 5. The C 1s peak region is fitted into three components for pDA-IONPs and GSSG-pDA-IONPs (Figure 5A,B). pDA shell on IONPs shows two dominant peaks attributed to C–C/C–H at 284.8 eV and C–N/C–O at 286.2 eV of the dopamine polymerized structure and one weak peak attributed to C=O at 288.4 eV that corresponds to the possible tautomers of pDA [44].

The incorporation of GSSG into the pDA shell reveals a slight shift in the peak position of C–N/C–O and O–C=O bonds to 285.8 and 288.1 eV, respectively, which suggests the immobilization of GSSG by introducing additional amine and carboxyl functional groups on pDA shell. To ensure the immobilization of GSSG, N (1s) peaks of the functionalized nanoparticles (Figure 5C,D) are fitted into two peaks at 398.4 eV for =N–R and at 400.1 eV for R–NH–R that correspond to the tertiary/aromatic and the secondary amines of dopamine intermediate structures [45,46]. The GSSG immobilization displays an additional peak corresponding to –NH_2_ at 402.1 eV, which is attributed to the free amine groups of glutathione structure [44]. Further, the XPS survey scan of GSSG-pDA-IONPs (Figure 4A) shows the presence of sulfur which confirms the immobilization of GSSG onto pDA-IONPs.

### 3.1. Reduction and S-nitrosation of GSSG-pDA-IONPs

**Figure 6:**
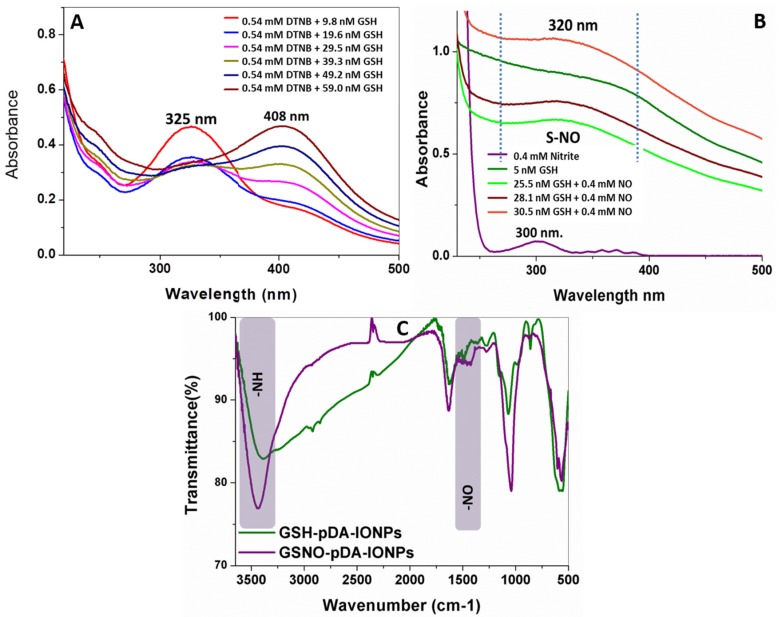
UV-visible spectra showing (**A**) the reduction of GSSG into GSH-pDA-IONPs; (**B**) S-nitrosation of GSH-pDA-IONPs; and (**C**) FTIR spectra of GSNO-pDA-IONPs and GSH-pDA-IONPs.

To serve as a dual modality to treat the cancer cells, GSSG-pDA-IONPs were first reduced to GSH using Elman’s reagent and then S-nitrosated using sodium nitrite. UV-visible spectra of reduced nanoparticles (Figure 6A), show a sharp shift in the absorbance peak from 325 to 408 nm on successive addition of GSSG-pDA-IONPs into 5,5-dithiobis-[2-nitrobenzoicacid] (DTNB) which confirms the presence of –SH in GSH-pDA-IONPs. Indeed, DTNB shows its characteristic absorbance band at 325 nm. When it reacts with the free sulfhydryl group in the reaction mixture, it gives a mixed disulfide or TNB (2-nitro-5-thiobenzoic acid), characterized by the absorbance band at 408 nm, which is in good agreement with the FTIR spectra (Figure 6C). The biological effect of S-nitrosothiol is attributed to the NO release from the breaking of the S–N bond to act as NO donor under physiological conditions. NO formation has the potential to influence the chemotherapy and contribute significantly to cancer biology [47]. Thus, with successive addition of GSH-pDA-IONPs in sodium nitrite solution, the formation of S-nitrosothiols is observed through the appearance of a broad and weak band at 320 nm (Figure 6B), which is in conformity with the weak absorbance shown by S-nitrosothiols [48].

The FTIR spectrum of GSNO-pDA-IONPs in Figure 6C shows the NO vibration band at ~1500 cm^−1^ which confirms the presence of S–NO in GSNO-pDA-IONPs. It is also shown that the broad band of GSH-pDA-IONPs, observed due to intramolecular hydrogen bonds, disappears on the formation of the S–NO bond, giving rise to the N–H bending band at 3440 cm^−1^ and suggesting the successful formation of S-nitrosothiols.

### 3.2. In-Vitro Drug Loading and Release Kinetics

DTX was first modified with PMPI prior to its loading and was characterized using FTIR and NMR (Figures S5 and S6). The hydroxyl group of DTX reacts with PMPI isocyanate and induces the disappearance of the isocyanate bond (Figure S5) and the formation of –CN, –NH and –CONH bonds, which are assigned respectively to 1310, 1520 and 1640 cm^−1^ vibration bands. This confirms the covalent bonding of DTX-PMPI, which was then loaded on GSH-pDA-IONPs. DOX- and DTX- loaded nanoparticles were further analyzed using DLS size measurement to ensure the stability of the nanocarrier in the biological medium (RPMI (Roswell Park Memorial Institute medium) and Albumin) at 37 °C where DTX-modified nanoparticles show an enhanced colloidal stability as compared to DOX-loaded nanoparticles (Figure S7).The PDI values of the nanocarrier dispersed in water were also measured where DOX-loaded nanoparticles had a value of 0.71 ± 0.24 and DTX-loaded 0.61 ± 0.10.

To evaluate the efficiency of the nanocarriers, it is very important to evaluate the drug loading efficiency of the designed drug delivery system. Since DOX is responsive in UV-vis spectroscopy due to its inherent chromophore, DOX loading and release kinetics were measured using both UV and TG analyses while quantification of loaded DTX was determined using TGA only. A comparative chart (Table S1, SI) shows the loading percentages of DOX and DTX analyzed using UV-vis spectroscopy and the High-performance liquid chromatography (HPLC) method, and observed in the literature.

**Figure 7:**
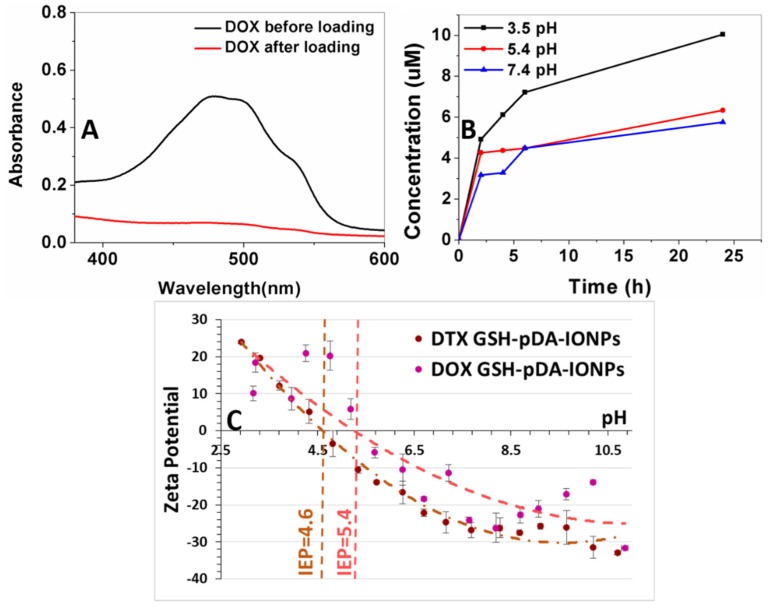
UV-vis absorbance spectra showing (**A**) doxorubicin loading on GSH-pDA-IONPs; (**B**) doxorubicin release at different pH in PBS (10 mM); and (**C**) zeta potential vs. pH curves of DOX- and DTX-drug loaded nanoparticles.

The surface loading efficiency of DOX, on the developed nanocarrier, is estimated to be 78% for 24 h of loading reaction (Figure 7A) using UV-vis absorbance measurement at 490 nm, which is the characteristic absorbance band of DOX molecule. The drug loading efficiency was calculated as given in Equation (1) [49].

(1) $$\%\;\mathrm{EE}=\frac{\mathrm{Concentration}\;(\mathrm{Drug}\;\mathrm{added}-\mathrm{Free}\;\mathrm{unentrapped}\;\mathrm{Drug}}{\mathrm{Concentration}\;\mathrm{of}\;\mathrm{Drug}\;\mathrm{Added}}\times 100$$

Drug release studies were carried out at three different pH (3.5, 5.4 and 7.4) with respect to the pH value of the endocytic compartment, which is lower than that of the normal physiological pH. Figure 7B shows that the release behavior of DOX from the GSH-pDA-IONPs is pH responsive because of the greater solubility of DOX in acid medium (low pH) [50]. This explanation is in good agreement with the results obtained from the zeta potential curves when measured at different pH (Figure 7C). Indeed, in acid medium, greater solubility and increased hydrophilicity of DOX is obtained due to the protonation of its –NH_2_ functional group [51]. The rate of DOX release is higher at low pH (3.5), but when the pH of the release medium increases to 5.4 and 7.4, the rate of the drug release decreases and the maximum release is observed during the initial 6 h as shown in Figure 7B.

Conclusively, moving from pH 7.4 to pH 3.5, the amount of released DOX has doubled. This could be due to the positively charged amine group and its effect on the drug’s solubility which depends on its protonation degrees [52]. Moreover, it has also been reported that at low pH condition, the ionization of the DOX molecule enhances its solubility which leads to an increased diffusion ability, preventing all non-bonding interactions with the polymeric layer of pDA [53]. On comparing the release profile at all pH, two stages of drug release are observed defining the DOX interaction: the first is between 0 and 2 h, where the drug release is fast and can be attributed to the adsorbed drug. The second stage is between 2 and 24 h, where the drug release is slow and could be attributed to the covalently linked drug. As the low pH favors the protonation of the DOX molecule, so the maximum drug release is observed at pH 3.5.

Thus, 78% of DOX loading can result in generating enough concentration of drug within a short span of time, thereby providing a great potential in developing an excellent nanocarrier and a positive approach towards an improved targeted cancer therapy.

**Figure 8:**
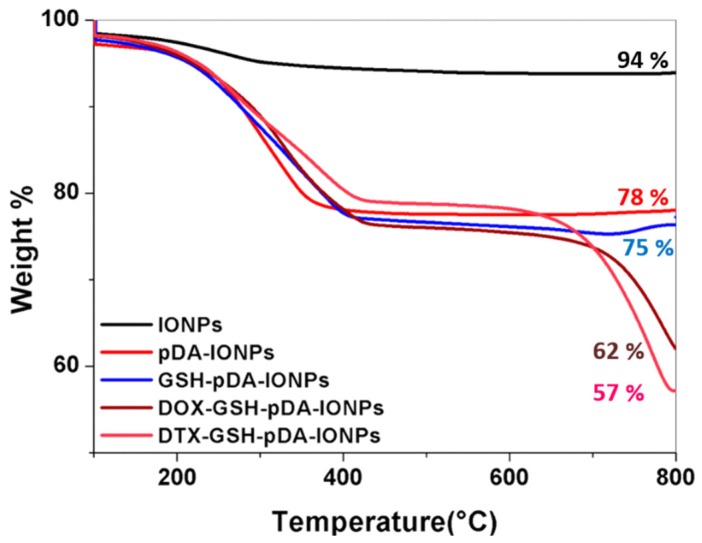
Thermogravimetric analysis (TGA) curves showing the sequential grafting of different organic molecules on the surface of IONPs.

TGA measurements (under air flow; nitrogen:oxygen 80:20), on the other hand, support quantifying the drug and immobilization steps. They indicate a weight loss of 6.1% (Figure 8) for bare IONPs, which later helps in the quantification of the organic coating. This weight loss, in the range of 100 to 800 °C, is due to the desorption of the physically adsorbed water and the dehydroxylation of hydroxyl groups on the surface. On heating pDA-IONPs, the weight loss increases to 22%, suggesting the degradation of the polymeric pDA coating on the surface. When GSH is immobilized on pDA-IONPs, the weight loss further increases to 25%, which corresponds to 97.6 µmol of GSH/g of IONPs. The amount of the loaded drug on GSH-pDA-IONPs is also calculated using TGA where 13.2% of weight loss is obtained for DOX and 18.0% for DTX. Drug-loading efficiency is then estimated to be 243 µmol/g for doxorubicin (DOX) and 223 µmol/g for docetaxel (DTX). The details of the analysis are presented in Table 2.

### 3.3. Cytotoxicity Assay

**Figure 9:**
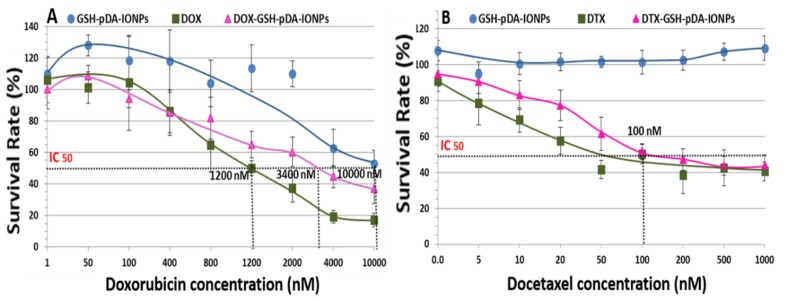
MTS ((3-(4,5-dimethylthiazol-2-yl)-5-(3-carboxymethoxyphenyl)-2-(4-sulfophenyl)-2H- tetrazolium)) assay showing the average percent of cell survival against various concentration of (**A**) DOX; and (**B**) DTX drug on PC3 cell lines when incubated for 48 h. (Standard deviations were calculated with six measurements).

In vitro cytotoxicity activity of DOX was assessed using MTS assay against PC3 cell lines where cells were exposed to the DOX-loaded nanoparticles for 48 h. The results obtained were preliminary but effective to explore the applicability of drug-loaded nanoparticles. Elevated dose studies were done with respect to the concentration of DOX as shown in Figure 9. The IC50 (the half maximal inhibitory concentration) value for DOX was reported to be 334.4–836.1 nM for 48 h [54,55]. On the other hand, our calculated IC50 value for the free drug is observed at 1,200 nM, which is increased to 3,400 nM when loaded on GSH-pDA-IONPs. It is also observed that the free drug is more toxic than the drug-loaded nanoparticles for maximum concentration. Indeed, the percentage of cell survival is 17.2 ± 4.3% for free DOX, while DOX-loaded nanoparticles show cell survival of 37.1 ± 9.5% in the studied range of concentration. This difference could be explained by the different behavior of the free and loaded DOX inside the cells [56,57]. DOX shows antiproliferative activity against many tumor cells and has the potential to permeate through the cell membrane very easily thanks to its hydrophobic nature. Unfortunately, DOX alone can also affect the growth of other healthy cells, leading to many side effects. These ultimately result in the depletion of the immune system and the human body becomes more prone to microbial infection, fatigue and, thus, the healing time decreases significantly [58]. Nanocarriers thus come into the role that can be used to target the cancer cells, thereby reducing the side effect of using a drug alone.

When MTS assay is performed with the DTX drug, the reported IC50 value is 11 nM [59]. However, in our case, the IC50 value of the loaded DTX drug increases to 100 nM due to the chemical modification of the molecule. This value is lower than that obtained by Loiseau et al. for DTX-modified titanate nanotubes where the obtained IC50 value was 390 nM [23]. Also, the percentage of surviving cells at a higher concentration is 43.9 ± 5.7% for DTX-loaded nanoparticles and 40.5 ± 5.2% for free DTX, as shown in Figure 9B. This suggests that it can be used as effective nanocarrier.

**Figure 10:**
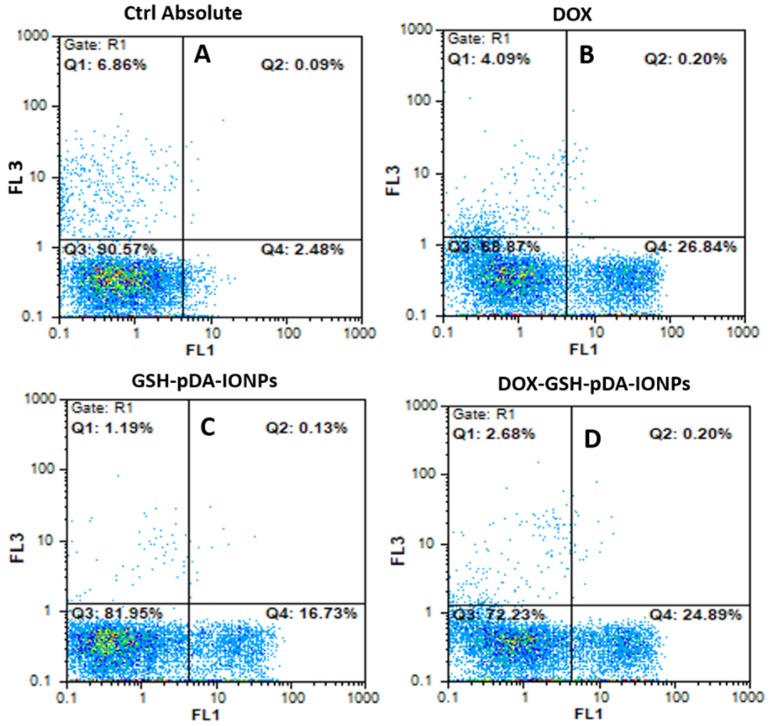
Representative dot plot of Annexin V-FITC/PI ((fluorescein isothiocyanate) detection kit with PI (propidium iodide) staining of PC3 (prostate cancer) cells incubated for 24 h A. Without any sample B. with DOX, C. GSH-pDA-IONPs and D. DOX-GSH-pDA-IONPs (flow cytometry: x axis (Fluorescence 1, FL1) Annexin V-FITC and y axis (FL3) propidium iodide). The Q1 quadrant represents unviable cells (PI positive and annexin negative). The Q2 quadrant represents cells that are in late apoptosis or necrosis (both annexin and PI positive). The Q3 quadrant represents viable cells (both annexin-FITC and PI negative). The Q4 quadrant represents cells in early apoptosis (annexin positive and PI negative).

The targeting specificity of the developed nanoparticles were further quantified using flow cytometry by an Annexin V-FITC detection kit with PI staining. Necrosis and apoptosis are measured as shown in Figure 10.

At an equivalent drug concentration where the concentration of only GSH-pDA-IONPs in the nanohybrid is in the range of 0.01 to 5.3 µg mL^−1^, it is observed that DOX-loaded nanoparticles reveal a slightly lower antitumor activity compared to free DOX. This may be due to the interaction mechanism of DOX-GSH-pDA-IONPs as it is observed in MTS and confocal studies (Figure S8) [60]. However, in this test, PC-3 cells were exposed to DOX for 24 h, whereas MTS assay cells were exposed for 48 h. On further analyzing the nature of cell apoptosis, it is observed that DOX-loaded nanoparticles show 24.9% of early apoptotic (Annexin positive / PI negative) for only 24 h of exposure compared to 16.7% for free drug nanoparticles (GSH-pDA-IONPs). These results suggest that even a small concentration is able to induce cell apoptosis, which confirms the antiproliferative effect of DOX-loaded nanoparticles.

## 4. Conclusions

In this work, we attempted to develop nanocarriers based on IONPs to treat prostate cancer. IONPs were first coated with a biocompatible polymer, pDA, which provides an anchor for further functionalization. Then, glutathione was successfully grafted onto pDA-IONPs in order to increase the efficacy of the developed nanocarrier thanks to its intrinsic biological properties. Before going to the biological assays, the developed nanocarriers were fully characterized using different techniques (TEM, XPS, XRD, TGA, FTIR, UV-vis, DLS and zeta potential measurements) to ensure the successive coating and immobilization of pDA and GSSG onto the IONPs’ surface. Two therapeutic molecules were studied in this work: DOX and DTX. These two anticancer drugs have been widely used in the treatment of cancer but they have several side effects when used alone. Thus, to overcome these limitations, we developed nanocarriers that can decrease these side effects. This was preliminarily proved with MTS and apoptosis studies, performed on PC3 cell lines. Drug release studies were also carried out with DOX-loaded nanoparticles to explore the interaction behavior of drugs in physiological and cancerous cell environments (imitated by an acid medium). Subsequently, the GSSG grafted onto pDA-IONPs was reduced to obtain free thiols on the surface, which provides an additional advantage to the nanocarrier by forming S-nitrosothiols. This could also be used as a NO donor agent and, thus, proves its worth in cancer treatments.

## Figures and Tables

**Table 1:** XPS elemental analysis (atomic percentage) of the prepared nanoparticles.

Samples	Fe	O	C	N	S	N/C	Fe/O	Fe/C
IONPs	38	57	3	1	–	0.3	0.7	13
pDA-IONPs	16	37	42	5	–	0.1	0.4	0.4
GSSG-pDA-IONPs	9	28	59	3	0.1	0.1	0.3	0.2

**Table 2:** Detailed analysis of the weight loss of synthesized nanoparticles and quantification of the grafted molecules.

Samples	Weight Loss %	Amount of Organic Content %	Amount of DOX/DTX Loaded (µmol g^−1^ of IONPs)
**IONPs**	6	0.0	–
**pDA-IONPs**	22	16	–
**GSH-pDA-IONPs**	25	19	–
**DOX-GSH-pDA-IONPs**	38	32	243
**DTX-GSH-pDA-IONPs**	43	37	223

## Supplementary Materials

The following are available online at http://www.mdpi.com/2079-4991/9/2/138/s1, Figure S1: XRD pattern of the bare IONPs (λ = 1.540598 Å), Figure S2: SAED patterns obtained via TEM analyses of A. bare IONPs and B. pDA-IONPs, Figure S3: Suspension images of (A). pDA-IONPs and (B). GSSG-pDA–IONPs after 24 h in PBS (10 mM, pH 7.4), Figure S4: Calibration curve showing the magnetic susceptibility vs. concentration of bare IONPs. Measurements were carried out in deionized water at pH 5.0–6.0, Figure S5: FTIR spectrum of the prepared DTX-PMPI, Figure S6: 1H-NMR predicted spectrum of DTX-PMPI, Figure S7: DLS size measurements to check the colloidal stability of the developed nanoparticles drug loaded FDG (GSH-pDA-SPIONs) in biological media (RPMI and albumin in NaCl) at 37 °C, Figure S8: Confocal images of PC3 cell lines with A. no treatment, B. only DOX and C. DOX loaded nanoparticles; Table S1: Comparative chart showing the drug loading efficiencies of doxorubicin and docetaxel, calculated using different method.
