# Supplementary material for: Polydopamine Modified Superparamagnetic Iron Oxide Nanoparticles as Multifunctional Nanocarrier for Targeted Prostate Cancer Treatment
Source: Nanomaterials (Basel). 2019 Jan 22;9(2):138. doi: 10.3390/nano9020138 (PMC6409598; doi:10.3390/nano9020138)
Supplement: Supplementary file 1 [file nanomaterials-09-00138-s001.pdf]

Supplementary Materials

Polydopamine Modified Superparamagnetic Iron Oxide Nanoparticles as Multifunctional Nanocarrier for Targeted Prostate Cancer Treatment

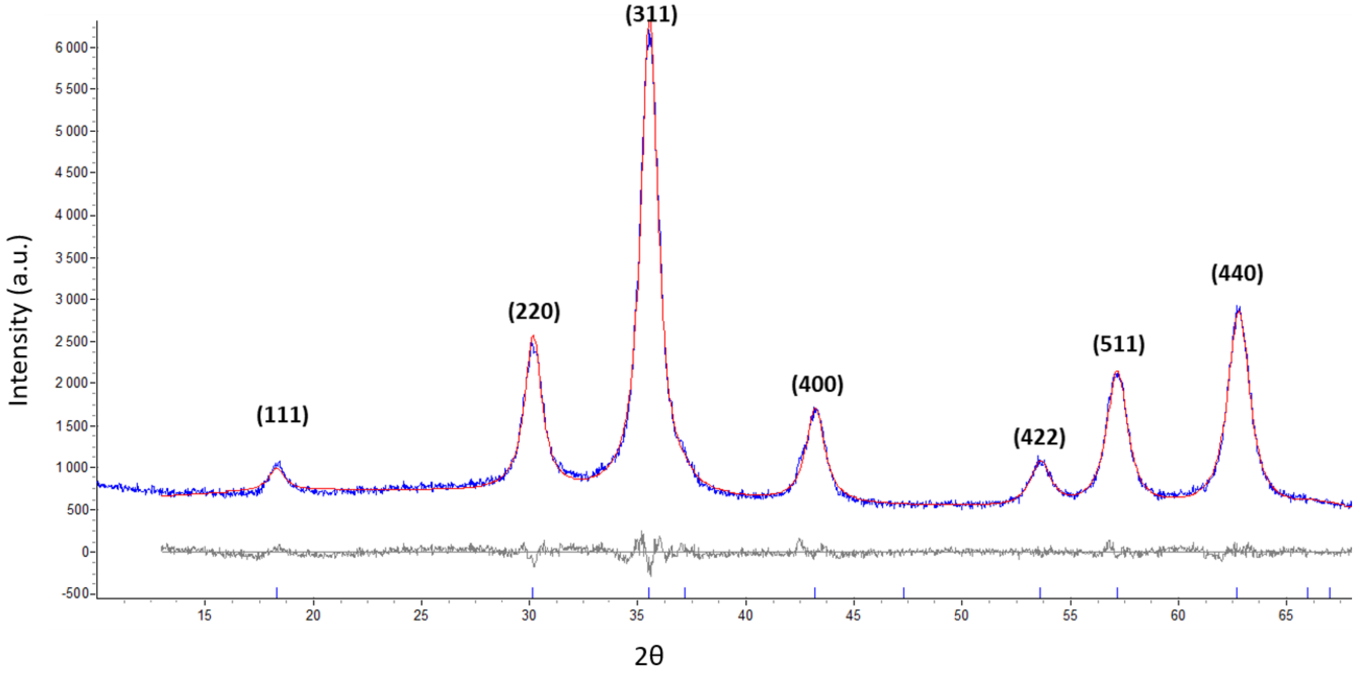

Figure S1. XRD pattern of the bare IONPs (λ = 1.540598 Å).

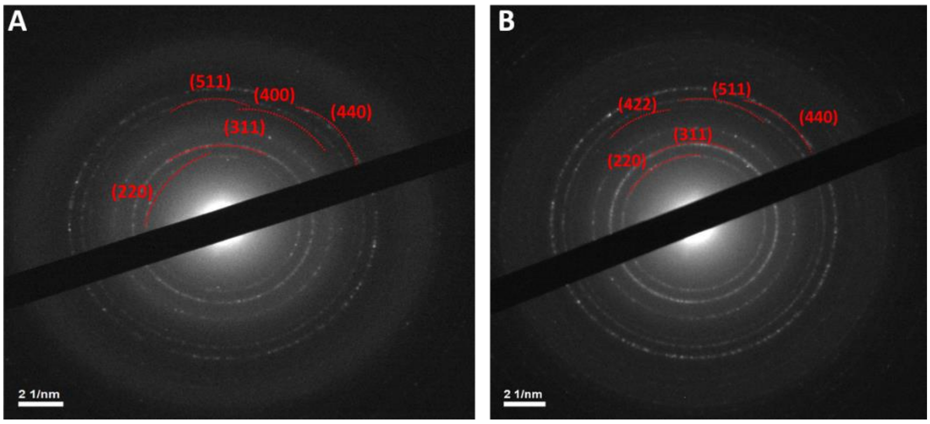

Figure S2. SAED patterns obtained *via* TEM analyses of A. bare IONPs and B. pDA-IONPs.

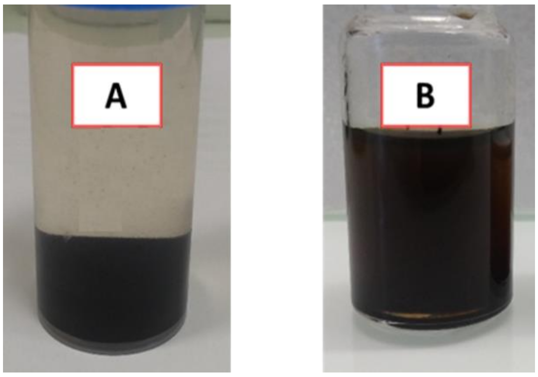

Figure S3. Suspension images of (**A**). pDA-IONPs and (**B**). GSSG-pDA–IONPs after 24 h in PBS (10 mM, pH 7.4)

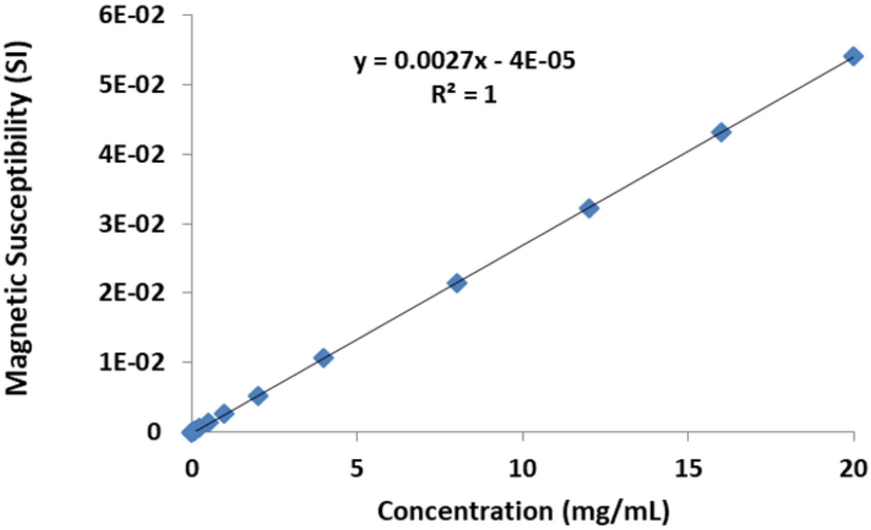

Figure S4. Calibration curve showing the magnetic susceptibility vs. concentration of bare IONPs. Measurements were carried out in deionized water at pH 5.0-6.0.

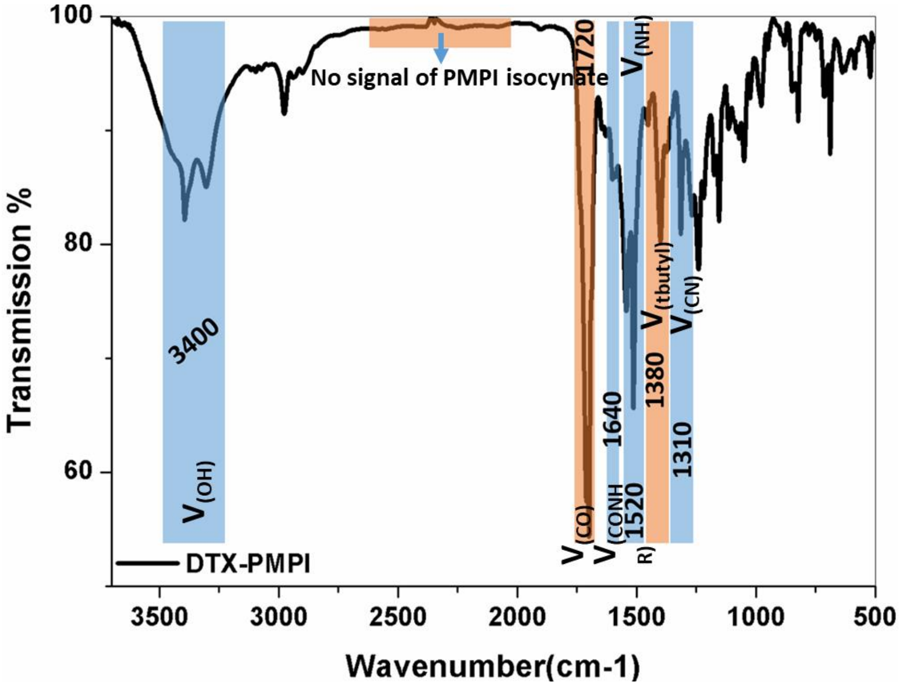

Figure S5. FTIR spectrum of the prepared DTX-PMPI.

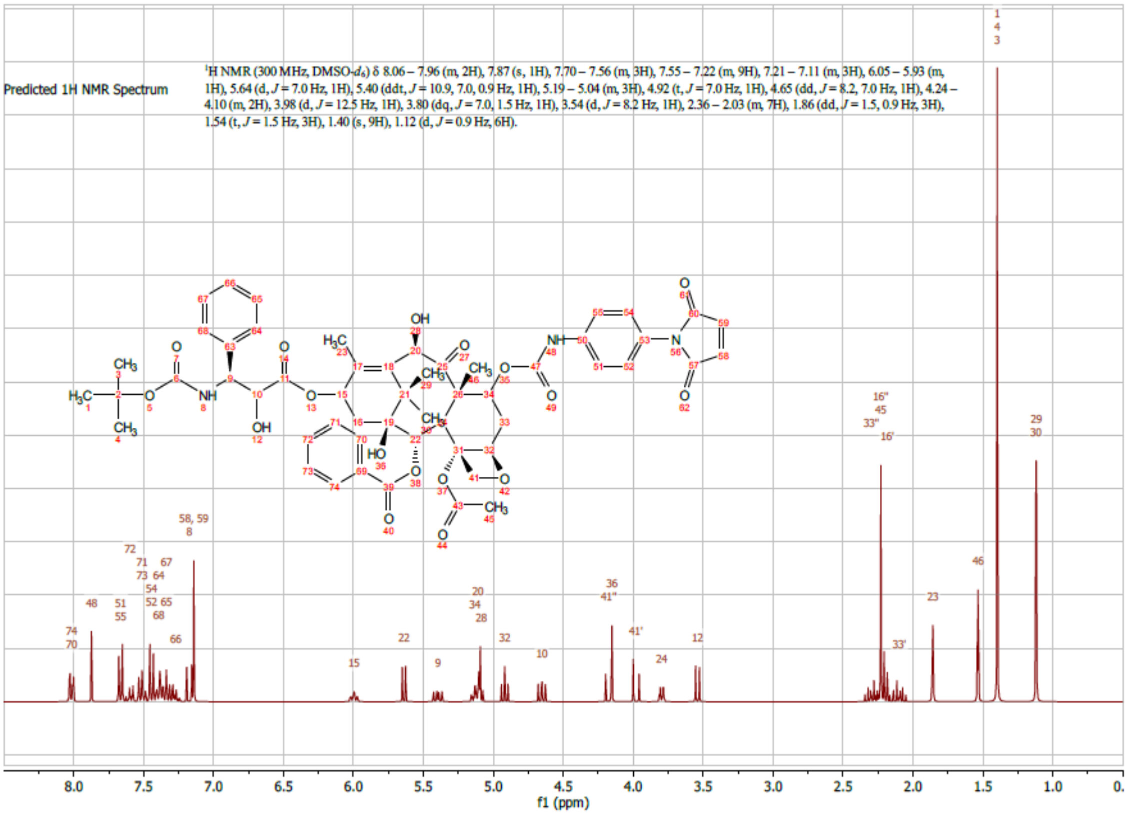

Figure S6. ^{1}H-NMR predicted spectrum of DTX-PMPI.

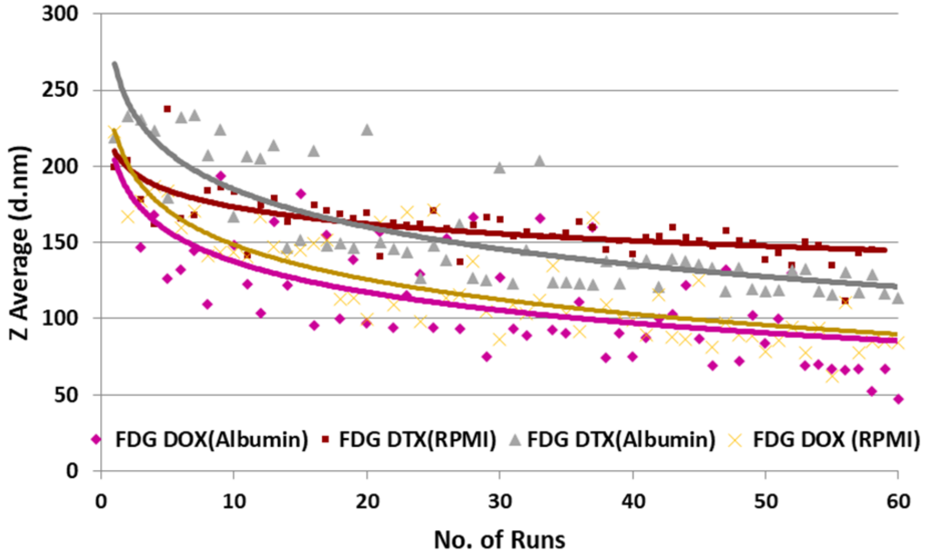

Figure S7. DLS size measurements to check the colloidal stability of the developed nanoparticles drug loaded FDG (GSH-pDA-SPIONs) in biological media (RPMI and albumin in NaCl) at 37 °C.

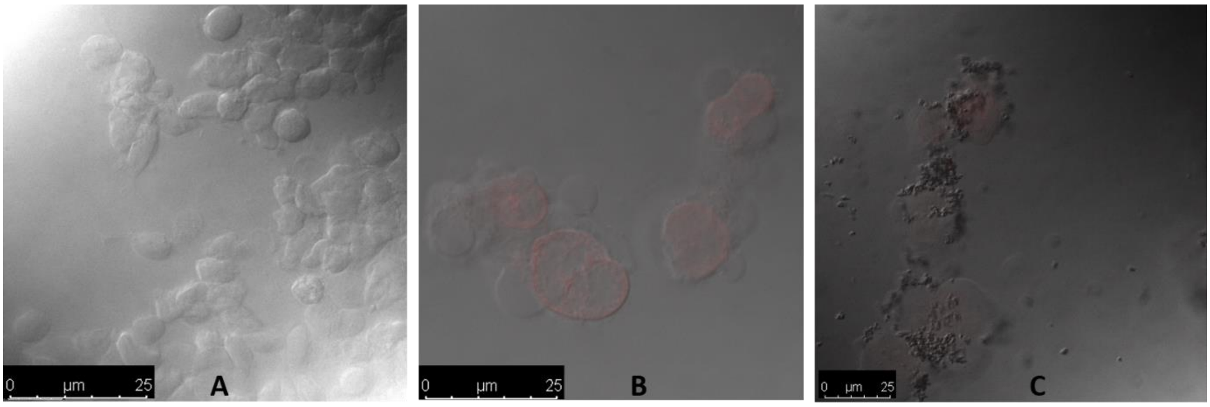

Figure S8. Confocal images of PC3 cell lines with A. no treatment, B. only DOX and C. DOX loaded nanoparticles.

Table S1. Comparative chart showing the drug loading efficiencies of doxorubicin and docetaxel, calculated using different method.

Sr. No	Loading Method (DOX)	Loading %	Loading Method (DTX)	Loading %
1.	UV-vis spectroscopy	< 50[1]	HPLC	~ 40 [2]
2.	UV-vis spectroscopy	68[3]	HPLC	9 [4]
3.	UV-vis spectroscopy	2.5 [5]	HPLC	67.5 [6]
4.	UV-vis spectroscopy	76[7]	HPLC	~ 23 [8]
5.	UV-vis spectroscopy	72 [9]	HPLC	52.7 [10]
6.	UV-vis spectroscopy	6.5 [11]	HPLC	~ 8 [12]
7.	UV-vis spectroscopy	62 [13]	HPLC	~8.9 [14]

References

1. Zhang, H.; Fei, J.; Yan, X.; Wang, A.; Li, J., Enzyme-Responsive Release of Doxorubicin from Monodisperse Dipeptide-Based Nanocarriers for Highly Efficient Cancer Treatment In Vitro. *Advanced Functional Materials* **2015,** *25* (8), 1193-1204.
2. Zhu, Y.; Zhang, J.; Meng, F.; Cheng, L.; Feijen, J.; Zhong, Z., Reduction-responsive core-crosslinked hyaluronic acid-b-poly(trimethylene carbonate-co-dithiolane trimethylene carbonate) micelles: synthesis and CD44-mediated potent delivery of docetaxel to triple negative breast tumor in vivo. *Journal of Materials Chemistry B* **2018,** *6* (19), 3040-3047.
3. Wang, X.; Sun, X.; Lao, J.; He, H.; Cheng, T.; Wang, M.; Wang, S.; Huang, F., Multifunctional graphene quantum dots for simultaneous targeted cellular imaging and drug delivery. *Colloids and Surfaces B: Biointerfaces* **2014,** *122*, 638-644.
4. Koopaei, M. N.; Khoshayand, M. R.; Mostafavi, S. H.; Amini, M.; Khorramizadeh, M. R.; Tehrani, M. J.; Atyabi, F.; Dinarvand, R., Docetaxel loaded PEG-PLGA nanoparticles: optimized drug loading, in-vitro cytotoxicity and in-vivo antitumor effect. *Iranian journal of pharmaceutical research: IJPR* **2014,** *13* (3), 819.
5. He, S.; Krippes, K.; Ritz, S.; Chen, Z.; Best, A.; Butt, H.-J.; Mailänder, V.; Wu, S., Ultralow-intensity near-infrared light induces drug delivery by upconverting nanoparticles. *Chemical Communications* **2015,** *51* (2), 431-434.
6. Wu, J.; Deng, C.; Meng, F.; Zhang, J.; Sun, H.; Zhong, Z., Hyaluronic acid coated PLGA nanoparticulate docetaxel effectively targets and suppresses orthotopic human lung cancer. *Journal of Controlled Release* **2017,** *259*, 76-82.
7. Thambi, T.; Deepagan, V. G.; Yoon, H. Y.; Han, H. S.; Kim, S.-H.; Son, S.; Jo, D.-G.; Ahn, C.-H.; Suh, Y. D.; Kim, K.; Chan Kwon, I.; Lee, D. S.; Park, J. H., Hypoxia-responsive polymeric nanoparticles for tumor-targeted drug delivery. *Biomaterials* **2014,** *35* (5), 1735-1743.
8. Sun, B.; Luo, C.; Li, L.; Wang, M.; Du, Y.; Di, D.; Zhang, D.; Ren, G.; Pan, X.; Fu, Q.; Sun, J.; He, Z., Core-matched encapsulation of an oleate prodrug into nanostructured lipid carriers with high drug loading capability to facilitate the oral delivery of docetaxel. *Colloids and Surfaces B: Biointerfaces* **2016,** *143*, 47-55.
9. Cao, X.; Tao, L.; Wen, S.; Hou, W.; Shi, X., Hyaluronic acid-modified multiwalled carbon nanotubes for targeted delivery of doxorubicin into cancer cells. *Carbohydrate Research* **2015,** *405*, 70-77.
10. Akhtartavan, S.; Karimi, M.; Karimian, K.; Azarpira, N.; Khatami, M.; Heli, H., Evaluation of a self-nanoemulsifying docetaxel delivery system. *Biomedicine & Pharmacotherapy* **2019,** *109*, 2427-2433.
11. Jang, H.; Kang, K.; El-Sayed, M. A., Facile size-controlled synthesis of fucoidan-coated gold nanoparticles and cooperative anticancer effect with doxorubicin. *Journal of Materials Chemistry B* **2017,** *5* (30), 6147-6153.
12. Li, L.; Cui, D.; Ye, L.; Li, Y.; Zhu, L.; Yang, L.; Bai, B.; Nie, Z.; Gao, J.; Cao, Y., Codelivery of salinomycin and docetaxel using poly (d, l-lactic-co-glycolic acid)-poly (ethylene glycol) nanoparticles to target both gastric cancer cells and cancer stem cells. *Anti-cancer drugs* **2017,** *28* (9), 989-1001.

13. Feng, Y.; Wang, C.; Ke, F.; Zang, J.; Zhu, J., MIL-100 (Al) Gels as an Excellent Platform Loaded with Doxorubicin Hydrochloride for pH-Triggered Drug Release and Anticancer Effect. *Nanomaterials (Basel, Switzerland)* **2018,** *8* (6).
14. Singh, R.; Norret, M.; House, M. J.; Galabura, Y.; Bradshaw, M.; Ho, D.; Woodward, R. C.; Pierre, T. G. S.; Luzinov, I.; Smith, N. M., Dose-Dependent Therapeutic Distinction between Active and Passive Targeting Revealed Using Transferrin-Coated PGMA Nanoparticles. *Small (Weinheim an der Bergstrasse, Germany)* **2016,** *12* (3), 351-359.
